# The spatial and temporal distribution of females influence the evolution of testes size in Australian rodents

**DOI:** 10.1098/rsbl.2022.0058

**Published:** 2022-05-04

**Authors:** Renée C. Firman, Dustin R. Rubenstein, Bruno A. Buzatto

**Affiliations:** ^1^ Centre for Evolutionary Biology, School of Biological Sciences (M092), The University of Western Australia, 35 Stirling Highway, Crawley, WA 6009, Australia; ^2^ Department of Ecology, Evolution and Environmental Biology, Columbia University, New York, NY 10027, USA; ^3^ College of Science and Engineering, Flinders University, Bedford Park, SA 5052, Australia; ^4^ Department of Natural Sciences, Macquarie University, Balaclava Road, Macquarie Park, NSW 2109, Australia

**Keywords:** sociality, post-mating sexual selection, sperm competition, mating season length, net primary productivity, testes size

## Abstract

Male–male competition after mating (sperm competition) favours adaptations in male traits, such as elevated sperm numbers facilitated by larger testes. Ultimately, patterns of female distribution will affect the strength of sperm competition by dictating the extent to which males are able to prevent female remating. Despite this, our understanding of how the spatial and temporal distributions of mating opportunities have shaped the evolutionary course of sperm competition is limited. Here, we use phylogenetic comparative methods to explore interspecific variation in testes size in relation to patterns of female distribution in Australian rodents. We find that as mating season length (temporal distribution of females) increases, testes size decreases, which is consistent with the idea that it is difficult for males to prevent females from remating when overlap among oestrous females is temporally concentrated. Additionally, we find that social species (spatially clustered) have smaller testes than non-social species (spatially dispersed). This result suggests that males may be effective in monopolizing reproduction within social groups, which leads to reduced levels of sperm competition relative to non-social species where free-ranging females cannot be controlled. Overall, our results show that patterns of female distribution, in both space and time, can influence the strength of post-mating sexual selection among species.

## 1. Background

Intrasexual competition over reproductive opportunities occurs in most species and typically among males that are limited by access to receptive females or resources linked to mating success [1]. At the premating level, agonistic interactions can lead to rapid evolutionary change in traits that are used in male contest [1]. However, male–male competition will extend beyond mating when females copulate with multiple males and the sperm of those males co-occur in the female reproductive tract [2]. This form of intrasexual selection, defined as sperm competition, is a pervasive selective force favouring adaptation in male reproductive anatomy and physiology [3,4], often via an increase in sperm production [5]. Indeed, larger relative testes size (RTS) across species often corresponds with evolutionary increases in the strength of selection from sperm competition [3]. For example, mammal species that have been subjected to a high level of sperm competition have larger RTS compared to those species where the strength of selection is reduced or absent [6–10].

Since differences among species in the level of sperm competition will, in part, depend on the ability of males to monopolize reproduction and prevent females from remating [11,12], the spatial distribution of sexually receptive females will influence male opportunity for reducing both the risk and intensity of sperm competition. In mammals, habitat and resource availability are important determinants of the spatial distribution of sexually receptive females, which in turn influence the spatial distribution of males because their fitness is dependent on the ability to find and defend mates [13,14]. Consequently, factors that influence female space use largely dictate social organization, and it is recognized that females of solitary species are typically more dispersed than those of social species, which are spatially clustered [14].

The degree of temporal clustering of female receptivity will also affect the level of sperm competition within species. A critical component to successful reproduction is producing young at a time when food resources are sufficient to ensure survival. Species develop adaptive responses to maximize their fitness according to the seasonal course of primary productivity, a major aspect of ecosystem functioning, within their range [15]. For example, large, mobile mammals may adapt their movements to spatio-temporal fluctuations in productivity, while smaller mammal species may match their breeding period to cycles of maximum vegetative growth [15]. In animals living in an environment with unpredictable food availability, the temporal pattern of breeding may result from the combined action of both environmental (spatial distribution of food resources) and social (pheromonal stimuli among grouped females) variables [16]. Irrespective of the underlying mechanism(s) that initiate reproduction, the duration of the mating season and consequently the extent to which female receptivity is temporally clustered, will influence the ability of individual males to monopolize individual females and prevent them from remating [17].

Like many other small mammal species, reproductive activity in Australian rodents coincides with high net-primary productivity (NPP) [15] when resources are sufficiently abundant [18]. Predictable seasonal patterns of cold winters in the south and summer rain in the north of Australia lead to relatively predictable seasonal breeding activity. For example, most northern species have been recorded breeding at all times of the year [18]. By contrast, erratic rainfall and sporadic resource availability in the arid zone lead to short, unpredictable periods of reproductive activity [18]. Moreover, a longitudinal study on two Australian desert rodent species revealed that reproductive activity was triggered by rain-induced seed availability [19]. Here, we explored the evolutionary association between the spatial and temporal distribution of oestrous females and the strength of sperm competition (as estimated by RTS) in Australian hydromyine rodents (Muridae). Rates of female remating and levels of sperm competition are expected to be greater among spatially clustered individuals of social species relative to solitary ones [14], and when mating seasons are relatively short and hence overlap among oestrous females is relatively high [20]. Alternatively, social species may experience lower levels of sperm competition than non-social species if individual males are able to control all reproduction within groups, for example by being socially dominant and evicting rivals or suppressing their reproductive attempts [21]. An alternative ‘temporal’ hypothesis predicts greater levels of sperm competition with increasing mating season length due to males being born, becoming sexually mature and breeding all within the same season [22].

## 2. Material and methods

### (a) Data collection

We used (i) *RTS* as an index for the strength of selection via sperm competition, (ii) *social organization* to represent a dichotomy in female spatial distribution and (iii) *mating season length* as a measure of the temporal distribution of female receptivity. Detailed information on data collection is provided in the electronic supplementary material. Briefly, male body and testes mass were obtained from a published source [23] or collected from specimens held in the Western Australian Museum collection (electronic supplementary material, table S1). Species were classified as ‘social’ (*n* = 16), based on evidence that individuals resided in groups or lived communally, or ‘non-social’ (*n* = 17), based on reports that individuals demonstrated behaviours reflective of a solitary existence [18] (electronic supplementary material, table S1). Finally, because individuals accrue fitness benefits by timing energy demands of reproduction to coincide with maximum food abundance, mating season length was estimated as the average number of months in a year for which NPP was positive [24] (electronic supplementary material, table S1). Since mating season length has the potential to vary across years as prevailing environmental conditions change, we calculated average NPP from 16 years of data and used the mean values in our analysis (details provided in the electronic supplementary material).

### (b) Data analysis

We based our inferences on a model selection approach [25], using the bias-corrected version of the Akaike information criterion (AIC*_c_*). We built a set of 11 candidate phylogenetic generalized linear models, with RTS as the response variable and every possible combination between the fixed effects of NPP, sociality (with the levels ‘social’ and ‘non-social’, as explained above) and average male mass (natural logarithm transformed), as well as two-way interactions between each pair of two of these variables (table 1). Data were manipulated and models were fit using functions of the package *caper* [26] in R v. 4.1.1. [27], and the molecular phylogeny was adapted from our previous study where we compiled available DNA sequences from five commonly sequenced genes, including a mitochondrial protein-coding locus (cytochrome b) and four nuclear exons (exon 11 of BRCA1, exon 10 of GHR, exon 1 of IRBP, and the single exon of RAG1) (see [18]). See the electronic supplementary material for more information on our statistical analyses.

**Table 1.** Model selection for the effects of NPP (a proxy for mating season length), social organization (S) and male BM on the RTS (a proxy for the level of sperm competition) of Australian rodents (*n* = 33 species).

N°	fixed effects	*k*	AIC*_c_*	ΔAIC*_c_*	weight	log-likelihoods	cumulative weight
1^a^	S + NPP + (S × NPP)	4	72.685	0.000	0.280	−31.628	0.280
2^a^	S + NPP	3	72.997	0.312	0.240	−33.085	0.520
*3^a^*	*NPP*	*2*	*74.052*	*1.367*	*0.142*	*−34.826*	*0.662*
4	BM + S + NPP	4	75.001	2.316	0.088	−32.786	0.750
5	S	2	75.592	2.907	0.066	−35.596	0.815
6	null	1	75.910	3.224	0.056	−36.890	0.871
7	BM + NPP	3	76.480	3.794	0.042	−34.826	0.913
8	BM + S	3	76.985	4.300	0.033	−35.079	0.946
9	BM + NPP + (BM × NPP)	4	77.611	4.926	0.024	−34.091	0.970
10	BM	2	78.134	5.449	0.018	−36.867	0.988
11	BM + S + (BM × S)	4	79.053	6.368	0.012	−34.812	1.000

^a^Indicates the most parsimonious models, and the simplest of these models is indicated in italics.

## 3. Results

**Figure 1.**
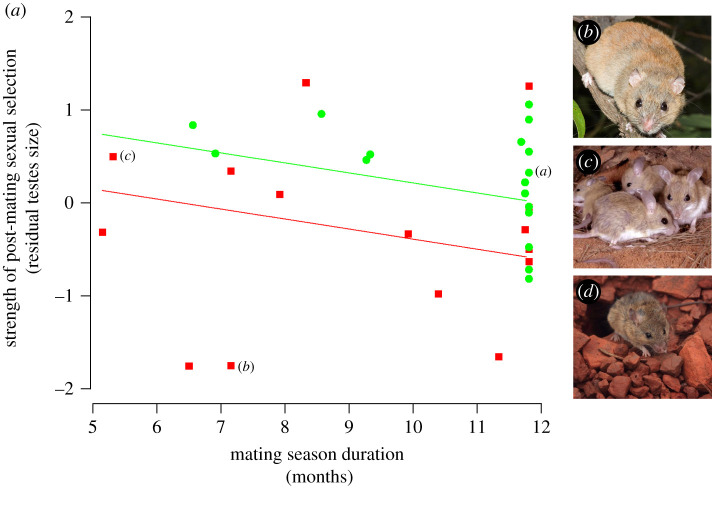
(*a*) The relationship between residual testes size, the length of the mating season (mean number of months of positive NPP) and social organization (red squares/line: social; green circles/line: non-social) in Australian rodents (*n* = 33 species). Predictions are based on a phylogenetic general linear model (number 2 in table 1). Australian rodent species discussed in the text: (*b*) fawn-footed mosaic-tailed rat (*Melomys cervinipes*; credit: Narelle Power), (*c*) spinifex hopping mouse (*Notomys alexis*; credit: Steve Parish) and (*d*) western pebble-mound mouse (*Pseudomys chapmani*; credit: Aline Gibson Vega). Images are the same as those presented in [18].

There was no single most parsimonious model describing variation in RTS across Australian rodent species; two models were within an AIC*_c_* of less than two from the best model (table 1). However, all three top models included a negative effect of NPP on RTS (with coefficients always significant and *p*-values lower than 0.029), and the relative importance (sum of AIC weights of all models including the variable) of this variable in our set of candidate models was 81.6%, strongly supporting that larger RTS was associated with shorter mating seasons (figure 1). Moreover, two of the top models included a negative effect of sociality on RTS, and the relative importance of this variable was 71.8%, revealing that social species have smaller RTS than non-social species (figure 1). Although the top model included a weak interaction between NPP and sociality with a relative importance of only 28% (table 1), the other two equally parsimonious models mentioned above did not.

## 4. Discussion

Interspecific differences in RTS were most closely related to the length of the mating season, a measure of the degree of oestrous synchrony among females. RTS was largest among species with the shortest mating seasons and declined as mating season length increased. We observed considerable variation in testes size among species that had the longest mating seasons (i.e. close to 12 months). In social species, this magnitude of variation was also seen in species with shorter mating seasons. This suggests that factors that we were unable to account for in our analysis may be influencing this result. For example, testes size is recognized to be a phenotypically plastic trait that changes according to the social environment that a male experiences during sexual development [28–31]. Unfortunately, we had no information on the developmental history of the individuals included in our study. Furthermore, our data were collected by multiple investigators, some species had small sample sizes and we were required to use mean values, with no measure of error around the data, in our analyses. Although it is possible that these limitations may have influenced our multispecies comparison, our analysis has revealed a general evolutionary pattern consistent with the idea that shorter periods of population-wide female sexual receptivity are expected to intensify male competition, since individual males cannot monopolize a series of receptive females but must instead compete at the same time if oestrus is synchronous across a population [20]. The relationship between mating season length and RTS that we report for Australian rodents is consistent with a previous study on semelparous marsupial species (those with male die-off), which showed that synchronized oestrus leads to high levels of sperm competition and increased female reproductive success [32]. Similar patterns have also been reported in comparative studies of birds and mammals where mating season length is correlated with the rate of extra-group paternity (i.e. the proportion of offspring sired by males that are external to the social group) [17,33]. These and other studies are consistent with the idea that synchronous breeding leads to resident males being unable to prevent females from engaging in extra-group copulations, which in turn would elevate both the risk and intensity of sperm competition [17,31,33–35].

In contrast with these previous comparative studies, our analysis indicated that males of social Australian rodent species may indeed be effective at monopolizing reproduction within groups. We found evidence that males of non-social species have larger RTS compared to social species, while controlling for mating season length, which suggests that the strength of selection via sperm competition acting on males is reduced in social species. An increase in the strength of post-mating sexual selection in non-social species is likely to be reflective of free-ranging individuals moving in and out of neighbouring territories and consequently the inability of males in preventing female remating. As an example, the Australian fawn-footed mosaic-tailed rat (*Melomys cervinipes*) is an arboreal, non-social species in which testes size equates to approximately 2% of the total body weight, and female home ranges overlap with multiple individuals [36]. Despite having smaller RTS overall, visualization of the data suggests that social species have more variation in RTS compared to non-social species. This pattern may be due to differences in the strength of selection from factors not included in our analysis. For example, non-social Australian rodents tend to live in relatively stable environments, which contrasts with social species that are more likely to occur in places with fluctuating or unpredictable environmental conditions [18]. Such disparity in stable versus unpredictable conditions may lead to a relatively consistent response to selection governed by the length of the mating season in non-social species, but not in social species.

In mammal social groups, a dominance hierarchy often regulates male access to receptive females or resources used to attract them, and typically males of high rank achieve the highest mating success [21]. In many species, juvenile males are evicted from the group by dominant adult males prior to reaching sexual maturity and posing as a threat as a reproductive rival [21]. In other cases, dominant males may tolerate the presence of subordinates, but with aggressive interactions often affecting the hormonal status of these potential rivals, for example by inducing low androgen levels and depressing their sexual behaviour [37]. As a result, some males remain in an adolescent-like state despite being able to reproduce. In other species, subordinate males have androgen levels that are comparable to those of dominant males but achieve low reproductive success because of behavioural mechanisms, such as being the recipients of targeted aggressive interactions during breeding periods [38]. Although sperm competition is common in Australian rodents [39,40], currently very little is known about the influence of social hierarchies on testes development and reproductive suppression in social groups. Interestingly, sexually mature males of the spinifex hopping mouse (*Notomys alexis*), a species that lives in mixed-sex groups in complex burrow systems, are reported to have full fertility potential despite having small testes and low sperm numbers [41].

Reproductive suppression can also occur through mate or resource monopolization. For example, dominant males suppress the reproductive success of subordinates by denying them access to females [21]. Similarly, monopolization of a critical resource for females, such as food or a breeding site, will inflict temporary reproductive suppression [21]. Australian western pebble-mound mice (*Pseudomys chapmani*) form social groups that work cooperatively to construct pebble mounds atop a subterranean burrow system [42,43]. Access to the pebble mound–burrow complex, which is a critical resource for breeding females, is likely to generate intense intrasexual competition among males. In this species, approximately 90% of males are described as being sexually mature but not bearing obvious testes (non-scrotal) [44], which raises the intriguing possibility that these males are reproductively suppressed subordinates, and the less abundant ‘scrotal’ males are dominant breeders. More research is required to elucidate how social hierarchies and mechanisms of reproductive suppression might be influencing the strength of post-mating sexual selection both within and among Australian rodent species.

In conclusion, our comparative study advances current knowledge on how the spatial and temporal distribution of mating opportunities influence the strength of post-mating sexual selection acting on males. On a temporal scale, our analysis suggests that shorter periods of population-wide female sexual receptivity intensifies sperm competition. Moreover, we provide novel evidence that males of social species (spatially clustered) have smaller testes than males of solitary, non-social species (spatially dispersed) potentially indicating that dominant males are effective at monopolizing reproduction within groups via mechanisms of reproductive suppression.

## Data Availability

The dataset supporting this article is available in the electronic supplementary material. Data were collected from the literature or from measurements taken from preserved specimens (details provided in the electronic supplementary material [45]).
